# Database-assisted screening of autism spectrum disorder related gene set

**DOI:** 10.1186/s13041-024-01127-0

**Published:** 2024-08-09

**Authors:** Éva Kereszturi

**Affiliations:** Department of Molecular Biology, Semmelweis University, Budapest, 1085 Hungary

**Keywords:** Autism spectrum disorder, ASD-related genes, Genetic variation, Syndromic ASD, Non-syndromic ASD, Gene set enrichment analysis, ASD-specific databases

## Abstract

Autism spectrum disorder (ASD) is a neurodevelopmental condition characterized by social and communication difficulties, along with repetitive behaviors. While genetic factors play a significant role in ASD, the precise genetic landscape remains complex and not fully understood, particularly in non-syndromic cases. The study performed an in silico comparison of three genetic databases. ClinVar, SFARI Gene, and AutDB were utilized to identify relevant gene subset and genetic variations associated with non-syndromic ASD. Gene set enrichment analysis (GSEA) and protein–protein interaction (PPI) network analysis were conducted to elucidate the biological significance of the identified genes. The integrity of ASD-related gene subset and the distribution of their variations were statistically assessed. A subset of twenty overlapping genes potentially specific for non-syndromic ASD was identified. GSEA revealed enrichment of biological processes related to neuronal development and differentiation, synaptic function, and social skills, highlighting their importance in ASD pathogenesis. PPI network analysis demonstrated functional relationships among the identified genes. Analysis of genetic variations showed predominance of rare variants and database-specific distribution patterns. The results provide valuable insights into the genetic landscape of ASD and outline the genes and biological processes involved in the condition, while taking into account that the study relied exclusively on in silico analyses, which may be subject to biases inherent to database methodologies. Further research incorporating multi-omics data and experimental validation is warranted to enhance our understanding of non-syndromic ASD genetics and facilitate the development of targeted research, interventions and therapies.

**Supplementary Information:**

The online version contains supplementary material available at 10.1186/s13041-024-01127-0.

## Background

Autism spectrum disorder (ASD) is a neurodevelopmental condition of varying severity with lifelong impact that can be recognized from early childhood and is characterized primarily by difficulties with social interaction and communication, and limited or repetitive patterns of thinking and behavior. Although its prevalence is estimated at 1%, it has been on a steadily increasing trend worldwide [1]. According to systematic public health data, the prevalence of ASD in the United States has increased from 1.47% to 2.76% in the last ten years [2, 3], but similar changes are also observed in Europe [4] and Asia [5]. The prevalence of autistic disorder is approximately four times higher in males than in females, and the gender differential is even higher in milder forms of ASD [6]. The hereditary nature of this condition is now a clear scientific fact, with some uncertainty about its exact extent. A meta-analysis found a heritability of 0.64‒0.91 [7], which has since been confirmed by others [8], giving a currently accepted heritability rate for this condition of 0.7‒0.8 [9]. A concordance of 98% for monozygotic twins and 53% for dizygotic twins has been found [7], and the sibling recurrence rate is estimated to be as high as 30% [10].

All these data point out a clear genetic predisposition, and the highly complex genetic nature of ASD is undeniable. To date, thousands of genetic variants in hundreds of genes have been identified, which can range from single nucleotide changes to the appearance of entire extra chromosomes, from rare mutations to very common polymorphisms, from de novo variants to hereditary ones [11]. Undoubtedly, most is known about the genetic background of ASD-associated syndromes with severe genetic abnormalities, which account for merely 20‒35% of all ASD cases. In contrast, in vast majority of individuals diagnosed with non-syndromic ASD, the genetic components are still largely unidentified [12].

The objective of this study is to conduct an in silico bioinformatics comparison between multiple ASD-specific genetic databases that are currently accessible online. Although these databases contain primarily genetic information related to severe syndromes, which often manifest differently and are largely associated with well-defined genetic anomalies, their overlap with prominent autistic symptoms may indicate a subset of genes specific to ASD and independent of the syndromic conditions. This gene set may serve as a valuable resource for increasing the efficiency of genetic targeting of the significantly more common non-syndromic ASD.

## Methods

### Data acquisition

Three databases were used to select the most relevant genes and genetic variations associated with ASD. ClinVar is a freely accessible, public archive of reports of human variations classified by diseases, in the present case ASD, together with supporting evidence ([13] https://www.ncbi.nlm.nih.gov/clinvar/ assessed on 10 May 2023). The SFARI Gene ([14] https://gene.sfari.org/ assessed on 24 May 2023) and AutDB ([15] http://autism.mindspec.org/autdb/Welcome.do assessed on 4 May 2023) are autism-specific databases in which risk genes are scored according to a set of strict annotation rules based on the evidence supporting their association with autism. For the exact process of gene selection, see the Results section. Variations in the selected genes were downloaded from the ClinVar and AutDB databases. In the case of ClinVar, in addition to the de novo mutations in the “Time of origin” category, variants with germline, maternal, and paternal inheritance were merged under the heading “familial”, as AutDB also distinguishes between these two groups. The number of affected genes was determined by examining the ClinVar records individually, whereas AutDB allows for the filtering of this parameter. The search for “molecular consequences” was conducted on the ClinVar interface, while the data downloaded from AutDB were filtered individually.

### Gene set enrichment analysis (GSEA)

The ShinyGO 0.77 tool [16] was employed to assess the correlation between the selected gene set and their biological function, as well as the functional network derived from the Gene Ontology (GO) database. OMIM disease data were also applied to assess the disease-relevance of the identified subset of genes. To increase reliability, the false discovery rate (FDR) threshold was reduced to 0.01 in all analyses, and only the first 10 significant hits selected by the FDR and sorted by fold enrichment (FE) were considered.

### Construction of protein–protein interaction (PPI) network

The PPI network of the ASD-related gene selection was generated and visualized using STRING 12.0 ([17] https://string-db.org/ assessed on 24 January 2024) with a minimum level of confidence < 0.4 to analyze the functional interactions among proteins.

### Statistics

Methods integrated into the software described above were used to statistically assess the integrity of the shared genes. Alterations in the distribution of genetic variations between databases were assessed using *χ*^2^-test for pairwise comparisons between genes. For multiple testing, Benjamini–Hochberg method was applied using sequential modified Bonferroni correction. Differences with a *p* < 0.05 value were considered to be statistically significant.

## Results

### Selection of relevant ASD-related genes

**Fig. 1:**
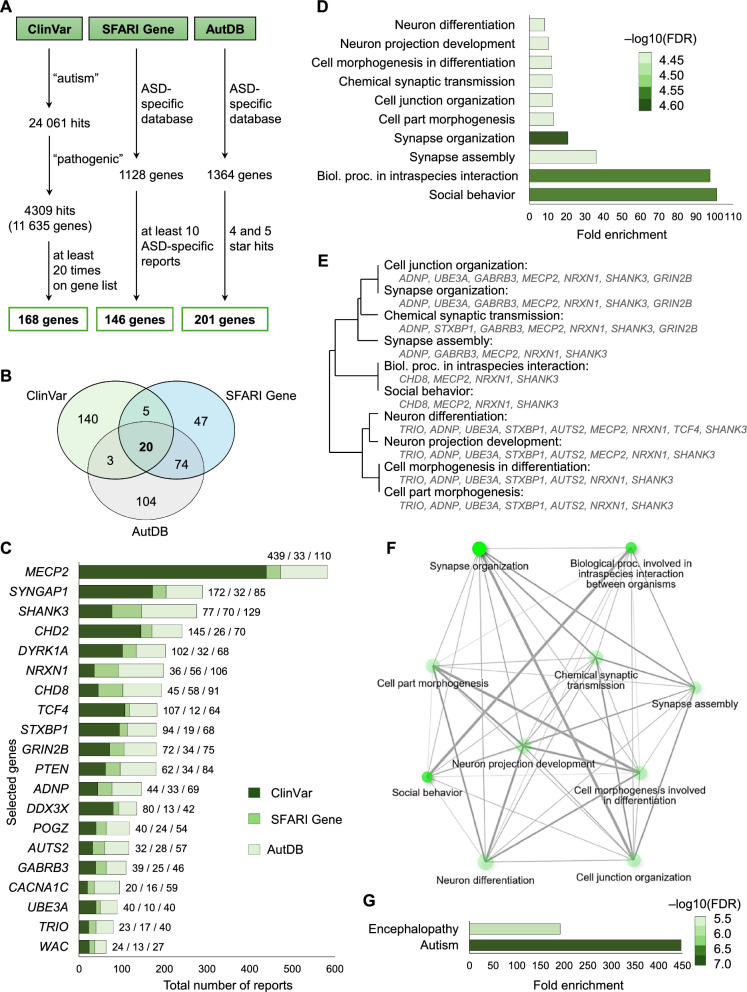
Screening of genes highly association with ASD and bioinformatic analysis of their biological interactions. **A** Search terms and sorting parameters with the number of hits in each stage for each database. **B** Venn diagram representation of overlapped genes. **C** Ranking of the 20 shared ASD-related genes based on the total number of reports from each database. The diagram depicts the overall ClinVar/SFARI Gene/AutDB report counts. GSEA of the 20 overlapping ASD genes for GOBPs (**D**), and their hierarchical clustering (**E**) and network analysis (**F**). The hierarchical clustering tree summarizes the correlation among significant pathways, with common genes clustered together. In network analysis two nodes are connected if they share 20% or more genes. Darker nodes are more significantly enriched gene sets. Bigger nodes represent larger gene sets. Thicker edges represent more overlapped genes. **G** GSEA of the 20 overlapping ASD genes for OMIM Disease database. For each GSEA performed, the FDR threshold was reduced to 0.01, and only the first 10 significant hits selected by the FDR and sorted by FE were considered

The screening process of the massive amount of ASD-specific genetic data from the three databases, the algorithm of the searches and the number of hits obtained in the different steps are summarized in Fig. 1A. The tens of thousands of hits for the search term “autism” in the ClinVar database were narrowed to those with pathogenic annotation, and then further processed with the 168 genes that were listed at least 20 times. At the time of the analysis SFARI Gene and AutDB contained 1128 and 1364 ASD-specific genes, respectively. In the former case, the 146 genes with at least 20 ASD-specific reports were considered. The latter database ranks the ASD relevance of genes on a 5-point scale, of which 201 genes with 4 and 5 asterisks meaning strong probable association were included in the present study. A total of 20 overlapping genes were identified in the comparison of the three independent hit lists (Fig. 1B). To determine the relative importance of the 20 powerful hits, all positive scientific reports were downloaded and summarized from all three databases (Fig. 1C). Interestingly, the citation order differed between databases, for instance, while *MEPC2* was most cited in ClinVar (439), AutDB (110) and the overall ranking (582), it was only in the middle range in SFARI Gene (33).

### GSEA of selected genes most relevant for ASD

In order to elucidate the biological role of the identified gene set, functional enrichment analysis was performed using ShinyGO 0.77 with GO terms. Regarding GO Biological Process (GOBP), “Social behavior” and “Biological processes in intraspecies interaction” were found the be the most enriched (101.2-fold and 97.5-fold) with the highest FDR (4.5 both) (Fig. 1D). Although “Synapse organization” only resulted in 20.8-fold enrichment, it was characterized by the highest FDR value (4.6) (Fig. 1D). The top 10 GOBPs also included “Synapse assembly”, “Cell part morphogenesis”, “Cell junction organization”, “Chemical synaptic transmission”, “Cell morphogenesis in differentiation”, “Neuron projection development” and “Neuron differentiation” with FDR values ranging from 4.4 to 2.3 (Fig. 1D). The hierarchical clustering tree summarizes the correlation among significantly enriched GOBPs (Fig. 1E). The analysis revealed two main clusters. One of them contained processes related to neuronal cell differentiation and is mainly hallmarked by the *TRIO* and *AUTS2* genes, the other could be further subdivided into two well-defined subclusters. While *GABRB3* was the common gene in the synaptic function group, the subcluster of social skills shared *CHD8*. The network analysis of the top 10 GOBPs revealed a well-defined, compact network containing all items (number of nodes = 10) with the maximum number of possible edges (9) for each member of the network, highlighting the close functional correlation between them (Fig. 1F). GSEA for the overlapping 20 genes was also performed from the perspective of disease based on OMIM data using ShinyGO 0.77. The analysis revealed only two conditions, of which “Autism” was characterized by an exceptionally high FE (447-fold) and FDR (6.9), and low *p*-value (1.2E−07) (Fig. 1G).

To validate the results presented above, GSEA was also performed separately on independent gene lists from the three databases used (see Fig. 1A). For the 168 genes from ClinVar, only three GOBPs were found to be common to the selected shared genes (Additional file 1: Fig. S1A). Although “Social behavior” also received the highest FE in this analysis, it was markedly lower than the value obtained for the shared gene list (17.9 vs 101.2) and had a much more modest FDR (2.5 vs 4.5). Hierarchical clustering identified less clear clusters (Additional file 1: Fig. S1B), and accordingly the network analysis of GOBPs suggested a network with only 9 nodes instead of maximal 10, with the number of edges varying between 0 and 7 (Additional file 1: Fig. S1C). The OMIM based GSEA only identified “Autism” with modest FE (63.3 vs 447) and FDR (3.7 vs 6.9) compared to the shared gene set (Additional file 1: Fig. S1D). Similar trends were observed when examining the gene list of the other two databases (Additional file 1: Fig. S2 and Additional file 1: Fig. S3). Two GOBPs overlapped with the shared gene set for SFARI Gene (Additional file 1: Fig. S2A) and only one for AutDB (Additional file 1: Fig. S3A), and none of them was “Social behavior”. Both were characterized by modest FEs coupled with relatively high FDRs (Additional file 1: Fig. S2A and Additional file 1: Fig. S3A), as well as uncertain hierarchical clusters (Additional file 1: Fig.S2B and Additional file 1: Fig. S3B) and incoherent networks (Additional file 1: Fig. S2C and Additional file 1: Fig. S3C) relative to the shared gene set. The OMIM-based GSEAs of the gene lists of both ASD-specific databases showed an enrichment of six clinical conditions that only partially overlapped (Additional file 1: Fig. S2B and Additional file 1: Fig. S3B). Although “Autism” had the highest FE value for the SFARI Gene, it was well below the value for the overlapping gene list (110.1 vs 447, Additional file 1: Fig. S2D), which was also observed for the analysis with AutDB data (39.8 vs 447, Additional file 1: Fig. S3D).

### In silico protein–protein interaction analysis

**Fig. 2:**
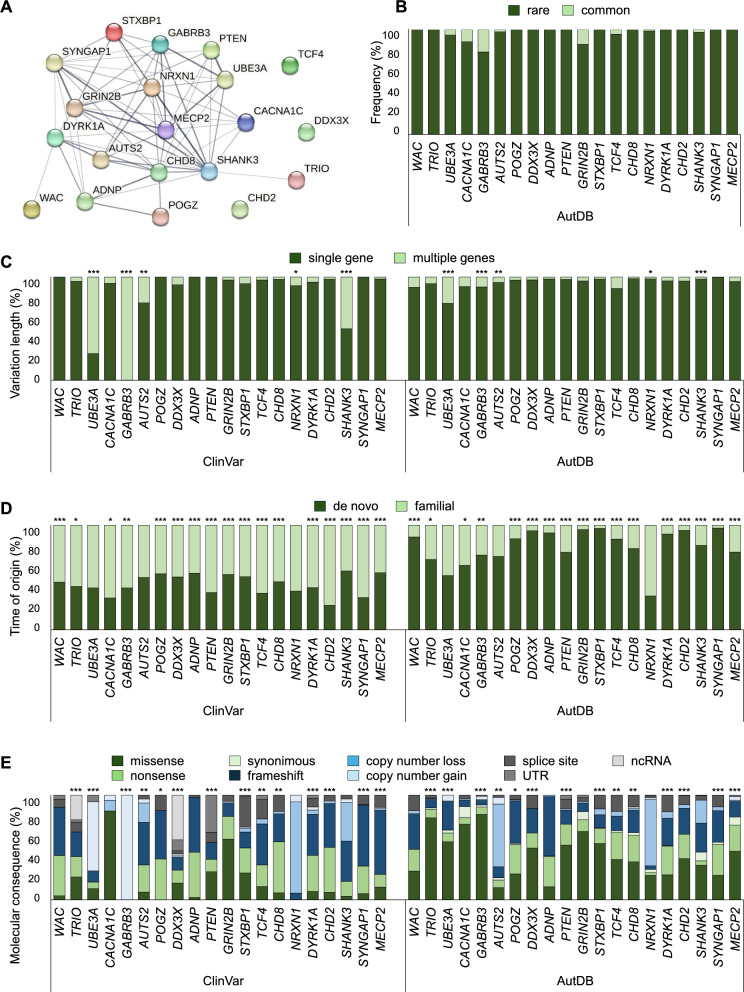
Examining PPI between members of the shared gene list, and comparing the distribution of differently classified genetic variants using ClinVar and AutDB data. **A** PPI network of the 20 shared ASD-specific proteins. Thicker edges represent more overlapped proteins. Distribution of genetic variant types of the 20 overlapping ASD genes by Frequency (**B**), Variation type (**C**), Time of origin (**D**) and Molecular consequence (**E**) based on ClinVar and AutDB. The distribution profiles of genetic variant type categories are represented on a percentage scale. For each gene, the total number of genetic variation types in a given category was considered to be 100% and their distribution was plotted. The significant adjusted *p*-values per gene between the two databases are indicated as follows: * *p* < 0.05; ** *p* < 0.01; *** *p* < 0.001

To further analyze the functional relationship between the shared 20 genes at the protein level, a protein–protein interaction network was also generated using STRING 12.0 (Fig. 2A). Of the 20 selected proteins, 17 contributed to the predicted PPI map with 64 edges and a PPI enrichment *p*-value of 1.0E-16, meaning that there are more interactions between proteins than would be expected for a random set of proteins of the same size and degree distribution drawn from the genome. This enrichment indicates that proteins as a group are most likely biologically related.

### Analysis of genetic variations in selected genes

Four (Frequency, Variation length, Time of origin and Molecular consequence) of the six dimensions of genetic variations associated with ASD and described elsewhere [11] were further analyzed for the 20 selected genes. The amount of genetic variation types in the different categories was downloaded from both the ClinVar and the AutDB databases. The “Frequency” dimension was an exception, as rare/common data could only be extracted from AutDB. In the “Frequency” category, rare variations were predominant for all 20 selected genes (Fig. 2B). The single-gene and multiple-gene affected variation types in the “Variation length” dimension showed significantly different patterns for variants in *UBE3A*, *GABRB3*, *AUTS2*, *NRXN1* and *SHANK3* genes (Fig. 2C). When comparing de novo and familial variations, all but three common ASD genes were revealed to have significantly different distribution profiles (Fig. 2D). While ClinVar contains a higher proportion of familial variations, AutDB is a collection of de novo mutations. Seven types of molecular consequences were also compared (Fig. 2E). Not surprisingly, significant distributional differences were detected in the majority of the 20 shared genes. The numbers of gene variations belonging to different genetic variation types, as well as the *p*- and adjusted *p*-values are given in Additional file 1: Tables S1, S2 and S3, respectively.

## Discussion

ASD presents a significant public health challenge, with increasing prevalence worldwide. This neurodevelopmental condition exhibits a complex etiology involving both genetic and environmental factors. Although understanding the genetic underpinnings of ASD would be crucial for developing targeted interventions and therapy, the genetic predisposing factors responsible for the condition have remained largely hidden despite many recent advances. Therefore, the aim of the present study was to compare ASD-specific genetic databases to identify shared genetic components associated with autism, independent of syndromic conditions, and elucidate their biological significance by in silico analysis.

The question is legitimately raised as to what is the point of searching for target genes in the era of whole genome sequencing, and even further narrowing down the list of them based on certain considerations. However, it must not be forgotten that the vast majority of information derived from sequencing data can only be accessed after appropriate bioinformatic analysis. Furthermore, to target relevant genetic information, it is necessary to know what to look for, and the ASD-specific gene list created in the present work can provide a useful and accurate tool for this purpose.

Undoubtedly, however, estimates of the number and composition of ASD-relevant genes vary widely among research groups, used databases, and clinical sequencing panels. A recent review of a gene set associated with autism and neurodevelopmental disorders (NDD) compiled a list of 83 high-confidence and NDD candidate genes using five disease-oriented databases. Remarkably, 14 of these were found to be in common with the 20 genes identified in the present work (*MECP2*, *WAC*, *GRIN2B*, *STXBP1*, *PTEN*, *TCF4*, *POGZ*, *DYRK1A*, *ADNP*, *AUTS2*, *CHD2*, *SYNGAP1*, *DDX3X*, *UBE3A*), but it should also be noted that, unlike the present study, they included cases of NDD, developmental disorder and intellectual disability, as a broader phenotype [18]. Another approach aimed to identify autism genes in the human genome based on patterns of gene–gene interactions and topological similarity of genes in the interaction network [19]. Using 760 autism-related genes from the SFARI Gene and OMIM databases as positive controls, all human genes were prioritized for ASD susceptibility. When comparing the first 50 hits, only three were found to be in common with those found in the present work (*WAC*, *NRXN1*, *UBE3A*).

In addition to the database analyses, some large exome sequencing studies have also been performed to refine the list of ASD predominant genes. While only four (*CHD8*, *PTEN*, *SHANK3*, *NRXN1*) of the 53 autism-related genes identified in one study were found to be common with our gene set [20], in another work, all 20 genes were present among their 381 hits [21]. However, whereas the former study worked exclusively with samples of ASD diagnosed individuals, the latter mainly examined NDD cases. Other large-scale whole-genome or exome sequencing studies of families with children affected by ASD have primarily focused on the role of rare inherited variants in the development of the condition. Ruzzo and colleagues identified 69 genes associated with ASD risk, including 24 that passed a stringent statistical correction [22]. It is noteworthy that there is a considerable degree of overlap (11 genes) between the gene list identified by the aforementioned study and the genes selected in the present work (*WAC*, *SHANK3*, *GRIN2B*, *POGZ*, *NRXN1*, *DYRK1A*, *CHD8*, *ADNP*, *CHD2*, *SYNGAP1*, *PTEN*). A comparable methodology has identified 72 genes linked to ASD, which is also in substantial concordance with our curated gene list (overlapping genes: *WAC*, *GRIN2B*, *STXBP1*, *CHD8*, *PTEN*, *SHANK3*, *POGZ*, *NRXN1*, *DYRK1A*, *ADNP*, *AUTS2*, *CHD2*, *SYNGAP1*) [23]. In contrast, other studies that also emphasized the role of rare genetic variants demonstrated no [24] or minimal [25] overlap (*SYNGAP1*) with the present study. It should be noted, however, that the latter researches were conducted with relatively smaller populations of a few tens of individuals.

The comparison of gene expression levels of ASD and control samples in different tissues may also open promising perspectives. Compared to an updated list of 109 genes found to be significantly dysregulated in individuals with autism from several recent ASD expression studies, merely one (*SHANK3*) was found to be shared with ours [26]. A further study, which is unique within the field, compared whole genome and RNA sequencing data from postmortem dorsolateral prefrontal cortex samples of nearly two hundred individuals across prenatal and postnatal development for various neuropsychiatric conditions, including ASD [27]. Of the 97 genes identified as ASD-related, 14 exhibited overlap with the gene set identified in the present study (*GABRB3*, *WAC*, *GRIN2B*, *STXBP1*, *CHD8*, *PTEN*, *TCF4*, *SHANK3*, *POGZ*, *NRXN1*, *DYRK1A*, *ADNP*, *CHD2*, *SYNGAP1*). Furthermore, nine of these exhibited alterations in expression across the temporal developmental scale delineated in the study, with three displaying an increasing trend (*WAC*, *STXBP1*, *SHANK3*) and six exhibiting a decreasing trend (*CHD8*, *TCF4*, *POGZ*, *NRXN1*, *DYRK1A*, *ADNP*). However, the Human Protein Atlas data indicate that the expression of all 20 genes we delineated was observed in the human cortex, with *STXBP1* exhibiting the highest expression, and only four genes (*GABRB3*, *STXBP1*, *GRIN2B*, and *NRXN1*) were specific to this tissue [28].

The partial overlap with the literature draws attention to the careful applicability of these databases, as they still contain subjective elements, both in the ranking algorithm of ASD-related genes included (which may vary significantly from database to database) and in the defining method of ASD phenotype and diagnosis [29].

The ASD phenotype is a well-defined common feature of several well-characterized genetic syndromes with quite diverse symptoms (e.g. Rett-, Fragile X- and Down syndrome, Neurofibromatosis, Tuberous sclerosis [11]). Accordingly, as in their phenotype, there is a probable overlap in their genotype as well, which was attempted to be identified in the presented work by analyzing and comparing in silico databases. The molecular biological and clinical examination of the relatively narrow set of genes and their variants thus mapped may actually bring researchers closer to elucidating the genetic predisposition of the non-syndromic cases that constitute the vast majority of ASD patients. In addition, the highly ASD related gene set selected in this work may provide guidance for the design of more targeted, population-based genetic screening tests in large samples by predicting genetic hotspots of the condition.

## Limitations

The study relied only on in silico analyses, which may be subject to database biases and limitations. Furthermore, these databases may occasionally overlap, while using very different algorithms to rank the role of a gene in ASD. The applicability of the narrowed list of shared genes to non-syndromic ASD is further limited by the fact that most of the genetic information currently available in ASD databases is linked to various severe syndromes. It is also indisputable that, similar to other in silico analyses, the screening conditions defined at the beginning of the study can always be considered subjective to a certain extent, and therefore may influence the final result to varying degrees.

## Conclusions

Overall, these findings contribute to our understanding of the genetic landscape of ASD and provide insights into potential molecular mechanisms underlying the disorder. The identified genes and enriched biological processes offer promising targets for further research and therapeutic development. However, it is essential to acknowledge the limitations of in silico analyses and the need for experimental validation to confirm the functional significance of the identified gene set. Moving forward, collaborative efforts integrating multi-omics data and leveraging advanced computational methodologies will be crucial for unraveling the complexities of ASD genetics. By elucidating the molecular basis of ASD, a significant step can be taken toward personalized interventions and improved outcomes for individuals affected by this condition.

### Supplementary Information

Additional file 1: Figure S1. GOBP and OMIM Disease GSEA of ASD-related genes selected exclusively from the ClinVar database, and hierarchical clustering and network analysis of enriched GOBPs. GSEA of the 168 ASD genes identified in ClinVar for GOBPs (A), and their hierarchical clustering (B) and network analysis (C). The hierarchical clustering tree summarizes the correlation among significant pathways. In network analysis two nodes are connected if they share 20% or more genes. Darker nodes are more significantly enriched gene sets. Bigger nodes represent larger gene sets. Thicker edges represent more overlapped genes. D GSEA of the 168 ASD genes identified in ClinVar for OMIM Disease database. For each GSEA performed, the FDR threshold was reduced to 0.01, and only the first 10 significant hits selected by the FDR and sorted by FE were considered. Figure S2. GOBP and OMIM Disease GSEA of ASD-related genes selected exclusively from the SFARI Gene database, and hierarchical clustering and network analysis of enriched GOBPs. GSEA of the 146 ASD genes identified in SFARI Gene for GOBPs (A), and their hierarchical clustering (B) and network analysis (C). The hierarchical clustering tree summarizes the correlation among significant pathways. In network analysis two nodes are connected if they share 20% or more genes. Darker nodes are more significantly enriched gene sets. Bigger nodes represent larger gene sets. Thicker edges represent more overlapped genes. D GSEA of the 146 ASD genes identified in SFARI Gene for OMIM Disease database. For each GSEA performed, the FDR threshold was reduced to 0.01, and only the first 10 significant hits selected by the FDR and sorted by FE were considered. Figure S3. GOBP and OMIM Disease GSEA of ASD-related genes selected exclusively from the AutDB database, and hierarchical clustering and network analysis of enriched GOBPs. GSEA of the 201 ASD genes identified in AutDB for GOBPs (A), and their hierarchical clustering (B) and network analysis (C). The hierarchical clustering tree summarizes the correlation among significant pathways. In network analysis two nodes are connected if they share 20% or more genes. Darker nodes are more significantly enriched gene sets. Bigger nodes represent larger gene sets. Thicker edges represent more overlapped genes. D GSEA of the 201 ASD genes identified in AutDB for OMIM Disease database. For each GSEA performed, the FDR threshold was reduced to 0.01, and only the first 10 significant hits selected by the FDR and sorted by FE were considered. Table S1. Case numbers of genetic variation types of the 20 shared genes for the “Variation length” dimension from ClinVar and AutDB, with *p* and adjusted *p*-values for the given genes. Adjusted *p*-values less than 0.05 are highlighted in bold italics. Table S2. Case numbers of genetic variation types of the 20 shared genes for the “Time of origin” dimension from ClinVar and AutDB, with *p* and adjusted *p*-values for the given genes. Adjusted *p*-values less than 0.05 are highlighted in bold italics. Table S3. Case numbers of genetic variation types of the 20 shared genes for the “Molecular Consequence” dimension from ClinVar and AutDB, with *p* and adjusted *p*-values for the given genes. Adjusted *p*-values less than 0.05 are highlighted in bold italics.

## Data Availability

The datasets analyzed during the current study are available from the corresponding author on reasonable request.

## Abbreviations

ASD: Autism spectrum disorder
FDR: False discovery rate
FE: Fold enrichment
GOBP: Gene Ontology: Biological Process
GSEA: Gene set enrichment analysis
NDD: Neurodevelopmental disorders
PPI: Protein–protein interaction
