# Supplementary material for: Database-assisted screening of autism spectrum disorder related gene set
Source: Mol Brain. 2024 Aug 9;17:55. doi: 10.1186/s13041-024-01127-0 (PMC11316361; doi:10.1186/s13041-024-01127-0)
Supplement: Supplementary file 1 — Additional file 1: Figure S1. GOBP and OMIM Disease GSEA of ASD-related genes selected exclusively from the ClinVar database, and hierarchical clustering and network analysis of enriched GOBPs. GSEA of the 168 ASD genes identified in ClinVar for GOBPs (A), and their hierarchical clustering (B) and network analysis (C). The hierarchical clustering tree summarizes the correlation among significant pathways. In network analysis two nodes are connected if they share 20% or more genes. Darker nodes are more significantly enriched gene sets. Bigger nodes represent larger gene sets. Thicker edges represent more overlapped genes. D GSEA of the 168 ASD genes identified in ClinVar for OMIM Disease database. For each GSEA performed, the FDR threshold was reduced to 0.01, and only the first 10 significant hits selected by the FDR and sorted by FE were considered. Figure S2. GOBP and OMIM Disease GSEA of ASD-related genes selected exclusively from the SFARI Gene database, and hierarchical clustering and network analysis of enriched GOBPs. GSEA of the 146 ASD genes identified in SFARI Gene for GOBPs (A), and their hierarchical clustering (B) and network analysis (C). The hierarchical clustering tree summarizes the correlation among significant pathways. In network analysis two nodes are connected if they share 20% or more genes. Darker nodes are more significantly enriched gene sets. Bigger nodes represent larger gene sets. Thicker edges represent more overlapped genes. D GSEA of the 146 ASD genes identified in SFARI Gene for OMIM Disease database. For each GSEA performed, the FDR threshold was reduced to 0.01, and only the first 10 significant hits selected by the FDR and sorted by FE were considered. Figure S3. GOBP and OMIM Disease GSEA of ASD-related genes selected exclusively from the AutDB database, and hierarchical clustering and network analysis of enriched GOBPs. GSEA of the 201 ASD genes identified in AutDB for GOBPs (A), and their hierarchical clustering (B) and networ [file 13041_2024_1127_MOESM1_ESM.pdf]

SUPPORTING INFORMATION

for

Database-assisted screening of autism spectrum disorder related gene set

Éva Keresszturi[1,*]

[1]Department of Molecular Biology, Semmelweis University, H-1085 Budapest, Hungary

[*]Corresponding author: Éva Keresszturi: kereszturi.eva@semmelweis.hu

Figure S1

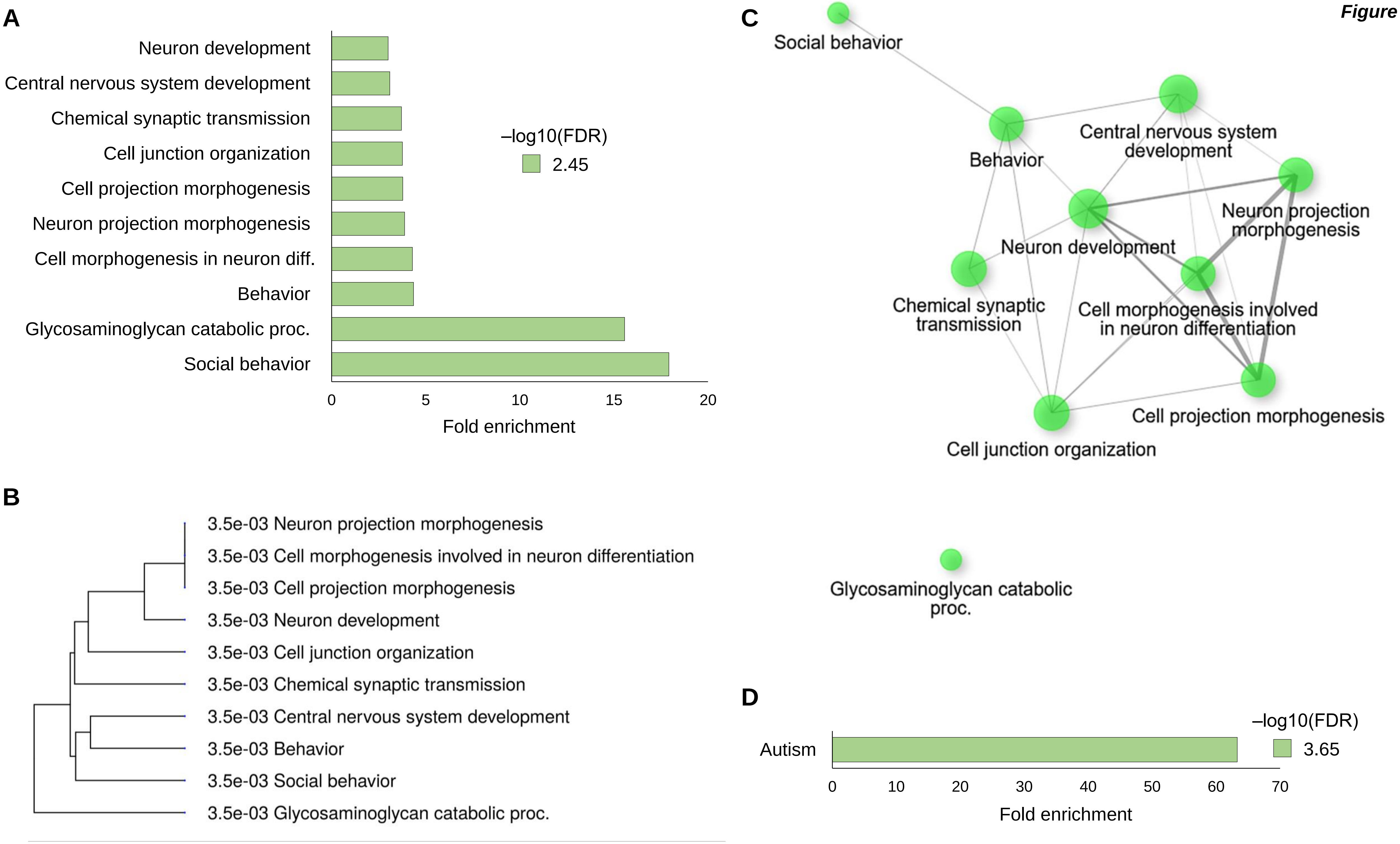
A
Neuron development
Central nervous system development
Chemical synaptic transmission
Cell junction organization
Cell projection morphogenesis
Neuron projection morphogenesis
Cell morphogenesis in neuron diff.
Behavior
Glycosaminoglycan catabolic proc.
Social behavior
–log10(FDR)
2.45
0
5
10
15
20
Fold enrichment
B
3.5e-03 Neuron projection morphogenesis
3.5e-03 Cell morphogenesis involved in neuron differentiation
3.5e-03 Cell projection morphogenesis
3.5e-03 Neuron development
3.5e-03 Cell junction organization
3.5e-03 Chemical synaptic transmission
3.5e-03 Central nervous system development
3.5e-03 Behavior
3.5e-03 Social behavior
3.5e-03 Glycosaminoglycan catabolic proc.
C
Social behavior
Central nervous system development
Behavior
Neuron projection morphogenesis
Neuron development
Chemical synaptic transmission
Cell morphogenesis involved in neuron differentiation
Cell projection morphogenesis
Cell junction organization
Glycosaminoglycan catabolic proc.
D
–log10(FDR)
Autism
3.65
0
10
20
30
40
50
60
70
Fold enrichment

Figure S2

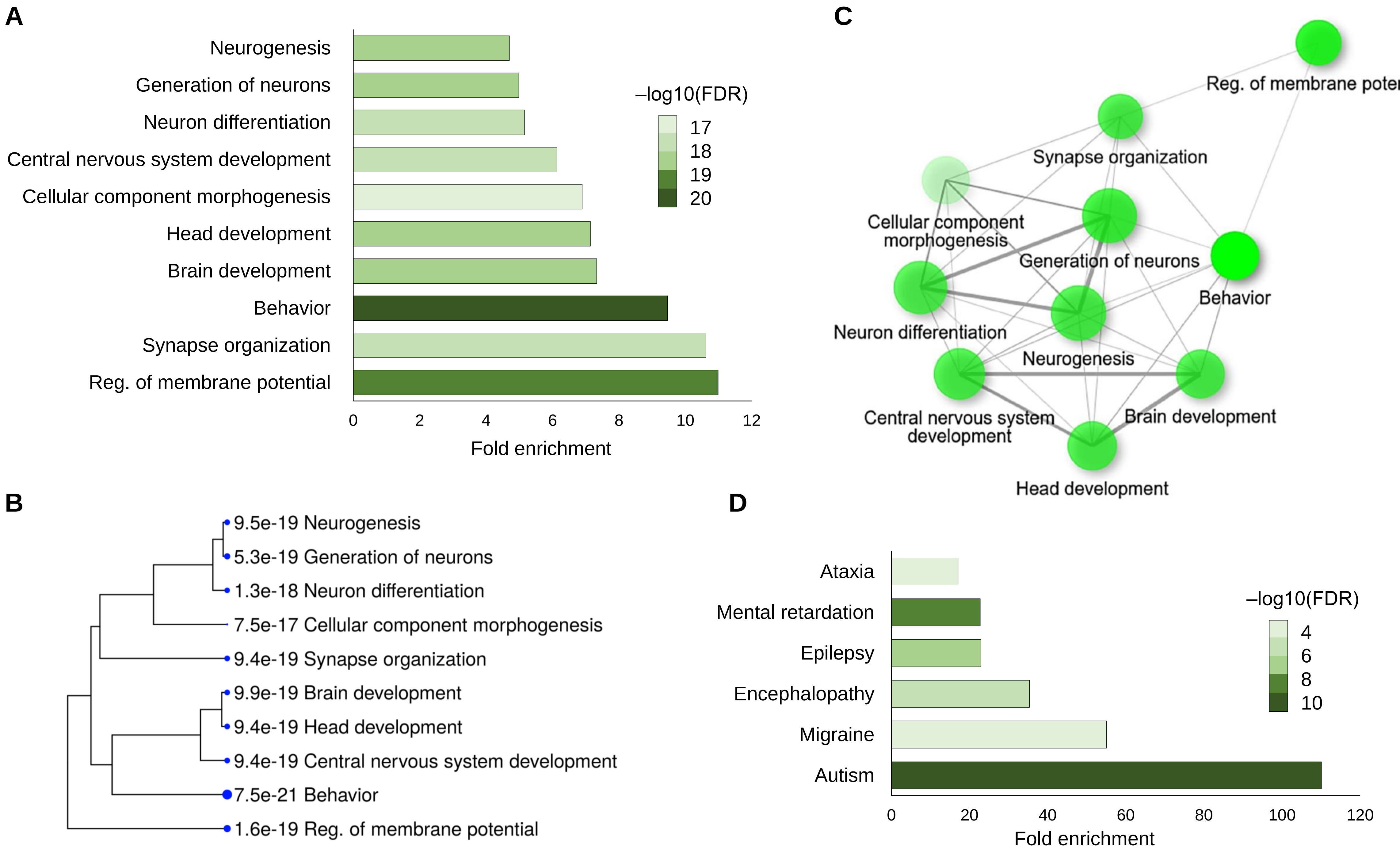
A
Neurogenesis
Generation of neurons
Neuron differentiation
Central nervous system development
Cellular component morphogenesis
Head development
Brain development
Behavior
Synapse organization
Reg. of membrane potential
–log10(FDR)
17
18
19
20
0
2
4
6
8
10
12
Fold enrichment
C
Reg. of membrane potential
Synapse organization
Cellular component morphogenesis
Generation of neurons
Behavior
Neuron differentiation
Neurogenesis
Central nervous system development
Brain development
Head development
B
9.5e-19 Neurogenesis
5.3e-19 Generation of neurons
1.3e-18 Neuron differentiation
7.5e-17 Cellular component morphogenesis
9.4e-19 Synapse organization
9.9e-19 Brain development
9.4e-19 Head development
9.4e-19 Central nervous system development
7.5e-21 Behavior
1.6e-19 Reg. of membrane potential
D
Ataxia
Mental retardation
Epilepsy
Encephalopathy
Migraine
Autism
–log10(FDR)
4
6
8
10
0
20
40
60
80
100
120
Fold enrichment

Figure S3

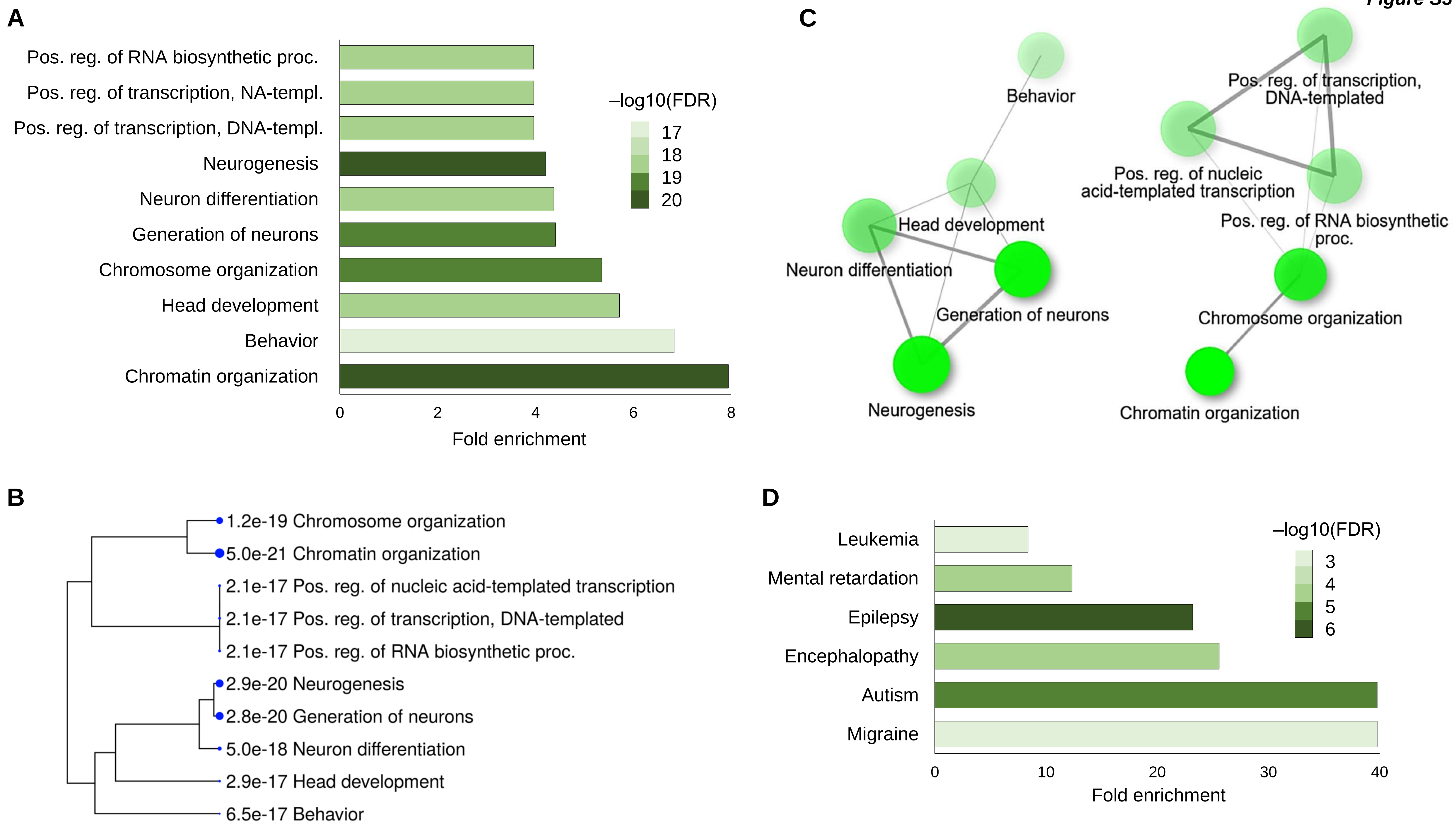
A
Pos. reg. of RNA biosynthetic proc.
Pos. reg. of transcription, NA-templ.
Pos. reg. of transcription, DNA-templ.
Neurogenesis
Neuron differentiation
Generation of neurons
Chromosome organization
Head development
Behavior
Chromatin organization
–log10(FDR)
17
18
19
20
0
2
4
6
8
Fold enrichment
B
1.2e-19 Chromosome organization
5.0e-21 Chromatin organization
2.1e-17 Pos. reg. of nucleic acid-templated transcription
2.1e-17 Pos. reg. of transcription, DNA-templated
2.1e-17 Pos. reg. of RNA biosynthetic proc.
2.9e-20 Neurogenesis
2.8e-20 Generation of neurons
5.0e-18 Neuron differentiation
2.9e-17 Head development
6.5e-17 Behavior
C
Behavior
Head development
Neuron differentiation
Generation of neurons
Neurogenesis
Pos. reg. of transcription, DNA-templated
Pos. reg. of nucleic acid-templated transcription
Pos. reg. of RNA biosynthetic proc.
Chromosome organization
Chromatin organization
D
Leukemia
Mental retardation
Epilepsy
Encephalopathy
Autism
Migraine
–log10(FDR)
3
4
5
6
0
10
20
30
40
Fold enrichment

Figure S1: GOBP and OMIM Disease GSEA of ASD-related genes selected exclusively from the ClinVar database, and hierarchical clustering and network analysis of enriched GOBPs. GSEA of the 168 ASD genes identified in ClinVar for GOBPs (**A**), and their hierarchical clustering (**B**) and network analysis (**C**). The hierarchical clustering tree summarizes the correlation among significant pathways. In network analysis two nodes are connected if they share 20% or more genes. Darker nodes are more significantly enriched gene sets. Bigger nodes represent larger gene sets. Thicker edges represent more overlapped genes. **D** GSEA of the 168 ASD genes identified in ClinVar for OMIM Disease database. For each GSEA performed, the FDR threshold was reduced to 0.01, and only the first 10 significant hits selected by the FDR and sorted by FE were considered.

Figure S2: GOBP and OMIM Disease GSEA of ASD-related genes selected exclusively from the SFARI Gene database, and hierarchical clustering and network analysis of enriched GOBPs. GSEA of the 146 ASD genes identified in SFARI Gene for GOBPs (**A**), and their hierarchical clustering (**B**) and network analysis (**C**). The hierarchical clustering tree summarizes the correlation among significant pathways. In network analysis two nodes are connected if they share 20% or more genes. Darker nodes are more significantly enriched gene sets. Bigger nodes represent larger gene sets. Thicker edges represent more overlapped genes. **D** GSEA of the 146 ASD genes identified in SFARI Gene for OMIM Disease database. For each GSEA performed, the FDR threshold was reduced to 0.01, and only the first 10 significant hits selected by the FDR and sorted by FE were considered.

Figure S3: GOBP and OMIM Disease GSEA of ASD-related genes selected exclusively from the AutDB database, and hierarchical clustering and network analysis of enriched GOBPs. GSEA of the 201 ASD genes identified in AutDB for GOBPs (**A**), and their hierarchical clustering (**B**) and network analysis (**C**). The hierarchical clustering tree summarizes the correlation among significant pathways. In network analysis two nodes are connected if they share 20% or more genes. Darker nodes are more significantly enriched gene sets. Bigger nodes represent larger gene sets. Thicker edges represent more overlapped genes. **D** GSEA of the 201 ASD genes identified in AutDB for OMIM Disease database. For each GSEA performed, the FDR threshold was reduced to 0.01, and only the first 10 significant hits selected by the FDR and sorted by FE were considered.

Variation length					
**Gene**	**Database**	**Singe gene**	**Multiple genes**	***p***	**adj_*p***
WAC	ClinVar	24	0	1.10E-01	2.20E-01
	AutDB	64	7		
TRIO	ClinVar	23	1	6.79E-01	7.54E-01
	AutDB	148	10		
UBE3A	ClinVar	14	40	6.47E-07	***4.31E-06***
	AutDB	38	13		
CACNA1C	ClinVar	46	3	5.28E-01	6.60E-01
	AutDB	79	8		
GABRB3	ClinVar	0	39	1.10E-29	***1.10E-28***
	AutDB	142	15		
AUTS2	ClinVar	24	8	6.40E-04	***3.20E-03***
	AutDB	111	6		
POGZ	ClinVar	40	0	2.76E-01	4.59E-01
	AutDB	201	6		
DDX3X	ClinVar	74	6	4.21E-02	1.05E-01
	AutDB	291	8		
ADNP	ClinVar	44	0	3.68E-01	5.25E-01
	AutDB	162	3		
PTEN	ClinVar	62	0	2.22E-01	4.03E-01
	AutDB	165	4		
STXBP1	ClinVar	70	2	6.99E-01	7.36E-01
	AutDB	232	9		
GRIN2B	ClinVar	89	6	2.58E-02	7.37E-02
	AutDB	308	6		
TCF4	ClinVar	104	3	2.10E-02	7.01E-02
	AutDB	72	9		
CHD8	ClinVar	44	1	7.67E-01	7.67E-01
	AutDB	305	5		
NRXN1	ClinVar	33	3	1.02E-02	***4.07E-02***
	AutDB	356	6		
DYRK1A	ClinVar	98	5	5.80E-01	6.82E-01
	AutDB	267	10		
CHD2	ClinVar	142	3	3.33E-01	5.12E-01
	AutDB	197	8		
SHANK3	ClinVar	39	39	1.08E-39	***2.16E-38***
	AutDB	402	8		
SYNGAP1	ClinVar	172	0	3.78E-01	5.04E-01
	AutDB	221	1		
MECP2	ClinVar	427	8	6.13E-02	1.36E-01
	AutDB	249	11		

Time of origin					
**Gene**	**Database**	**de novo**	**familial/ germline**	***p***	**adj_*p***
WAC	ClinVar	15	18	4.18E-05	***6.44E-05***
	AutDB	39	5		
TRIO	ClinVar	12	17	1.56E-02	***1.83E-02***
	AutDB	53	26		
UBE3A	ClinVar	8	12	4.21E-01	4.43E-01
	AutDB	14	13		
CACNA1C	ClinVar	7	16	1.29E-02	***1.61E-02***
	AutDB	32	20		
GABRB3	ClinVar	8	12	6.81E-03	***9.08E-03***
	AutDB	70	28		
AUTS2	ClinVar	13	13	6.86E-02	7.63E-02
	AutDB	49	21		
POGZ	ClinVar	30	26	2.20E-07	***4.40E-07***
	AutDB	134	20		
DDX3X	ClinVar	51	50	3.59E-23	***1.80E-22***
	AutDB	249	14		
ADNP	ClinVar	34	29	3.28E-09	***8.21E-09***
	AutDB	100	8		
PTEN	ClinVar	32	58	5.19E-08	***1.15E-07***
	AutDB	80	28		
STXBP1	ClinVar	57	51	1.61E-15	***5.36E-15***
	AutDB	134	6		
GRIN2B	ClinVar	66	64	1.83E-26	***1.22E-25***
	AutDB	226	7		
TCF4	ClinVar	45	84	3.36E-11	***9.60E-11***
	AutDB	52	8		
CHD8	ClinVar	28	33	2.63E-06	***4.78E-06***
	AutDB	146	42		
NRXN1	ClinVar	10	17	6.28E-01	6.28E-01
	AutDB	68	142		
DYRK1A	ClinVar	54	80	2.67E-22	***1.07E-21***
	AutDB	162	15		
CHD2	ClinVar	40	131	1.52E-39	***1.52E-38***
	AutDB	151	8		
SHANK3	ClinVar	32	25	1.53E-04	***2.19E-04***
	AutDB	166	40		
SYNGAP1	ClinVar	64	144	3.35E-40	***6.71E-39***
	AutDB	170	5		
MECP2	ClinVar	183	153	1.29E-05	***2.16E-05***
	AutDB	132	46		

		Molecular consequence										
**Gene**	**Database**	**missense**	**nonsense**	**synonimous**	**frame shift**	**copy number loss**	**copy number gain**	**splice site**	**UTR**	**ncRNA**	***p***	**adj_*p***
WAC	ClinVar	1	10	0	12	0	0	2	1	0	1.62E-01	1.80E-01
	AutDB	17	13	0	21	2	0	9	0	0		
TRIO	ClinVar	28	25	0	30	0	0	12	3	30	2.33E-19	***1.17E-18***
	AutDB	116	12	2	12	1	1	4	0	0		
UBE3A	ClinVar	5	3	0	5	0	31	0	3	0	4.17E-06	***9.26E-06***
	AutDB	20	3	1	10	0	2	0	0	0		
CACNA1C	ClinVar	17	0	0	0	2	1	0	0	0	1.55E-01	1.83E-01
	AutDB	47	4	7	4	1	0	2	0	0		
GABRB3	ClinVar	0	0	0	0	0	31	0	0	0	5.68E-21	***3.79E-20***
	AutDB	80	7	2	4	1	3	1	0	0		
AUTS2	ClinVar	2	7	0	11	5	1	1	0	0	1.07E-03	***1.78E-03***
	AutDB	11	7	2	10	57	6	2	0	0		
POGZ	ClinVar	0	16	0	19	0	0	6	0	0	1.34E-02	***1.79E-02***
	AutDB	49	56	2	80	2	1	8	0	0		
DDX3X	ClinVar	26	20	0	20	2	3	5	17	69	6.85E-39	***6.85E-38***
	AutDB	134	39	0	64	0	0	34	0	0		
ADNP	ClinVar	1	19	0	23	0	0	1	0	0	2.41E-01	2.54E-01
	AutDB	20	46	1	91	2	0	0	0	0		
PTEN	ClinVar	25	11	0	15	0	0	9	33	0	5.14E-08	***1.47E-07***
	AutDB	81	31	0	21	2	0	14	6	0		
STXBP1	ClinVar	27	25	0	16	4	1	31	1	0	3.34E-01	***6.07E-14***
	AutDB	134	23	16	17	3	0	7	5	0		
GRIN2B	ClinVar	40	15	0	9	0	1	4	0	0	1.52E-14	3.34E-01
	AutDB	161	43	2	37	17	2	37	0	0		
TCF4	ClinVar	13	21	0	40	5	0	19	4	0	1.46E-03	***2.25E-03***
	AutDB	25	17	1	9	3	0	10	0	0		
CHD8	ClinVar	3	22	0	12	1	0	7	0	0	6.47E-03	***9.24E-03***
	AutDB	109	76	8	64	6	4	33	0	0		
NRXN1	ClinVar	0	0	0	2	27	2	0	0	0	4.33E-02	5.41E-02
	AutDB	82	9	11	12	222	6	6	2	0		
DYRK1A	ClinVar	8	34	0	39	4	3	8	3	0	3.54E-05	***6.44E-05***
	AutDB	62	71	1	68	16	0	41	0	0		
CHD2	ClinVar	9	54	0	53	3	1	6	0	0	3.80E-08	***1.27E-07***
	AutDB	76	45	0	48	7	0	16	1	0		
SHANK3	ClinVar	2	8	0	22	21	2	2	0	0	6.49E-06	***1.30E-05***
	AutDB	126	16	32	105	84	3	14	1	0		
SYNGAP1	ClinVar	10	45	0	99	1	0	15	0	0	5.54E-07	***1.38E-06***
	AutDB	49	62	4	63	8	0	23	0	0		
MECP2	ClinVar	49	50	0	249	6	5	47	0	0	3.62E-44	***7.24E-43***
	AutDB	109	59	18	35	3	8	0	3	0		

Table S1: Case numbers of genetic variation types of the 20 shared genes for the "Variation length" dimension from ClinVar and AutDB, with *p* and adjusted *p*-values for the given genes. Adjusted *p*-values less than 0.05 are highlighted in bold italics.

Table S2: Case numbers of genetic variation types of the 20 shared genes for the "Time of origin" dimension from ClinVar and AutDB, with *p* and adjusted *p*-values for the given genes. Adjusted *p*-values less than 0.05 are highlighted in bold italics.

Table S3: Case numbers of genetic variation types of the 20 shared genes for the "Molecular Consequence" dimension from ClinVar and AutDB, with *p* and adjusted *p*-values for the given genes. Adjusted *p*-values less than 0.05 are highlighted in bold italics.
